# Establishment of an Individual-Specific Nomogram for Predicting the Risk of Left Ventricular Hypertrophy in Chinese Postmenopausal Hypertensive Women

**DOI:** 10.3390/medicina59030598

**Published:** 2023-03-17

**Authors:** Ruowen Yuan, Jianshu Chen, Shangyun Zhang, Xiaowei Zhang, Jing Yu

**Affiliations:** Department of Cardiology, Lanzhou University Second Hospital, Lanzhou 730030, China

**Keywords:** hypertension, postmenopausal, left ventricular hypertrophy, prognostic nomogram

## Abstract

*Background and Objectives:* The physiological phenomenon peculiar to women, namely menopause, makes the occurrence of left ventricular hypertrophy (LVH) in postmenopausal hypertensive women more characteristic. Less is known about the risk of developing LVH in Chinese postmenopausal hypertensive women. Thus, the present study was intended to design a nomogram for predicting the risk of developing LVH in Chinese postmenopausal hypertensive women. *Materials and Methods:* Postmenopausal hypertensive women aged between 49 and 68 years were divided into either the training set (*n* = 550) or the validation set (*n* = 284) in a 2:1 ratio. Patients in the validation set were followed up for one year. A stepwise multivariable logistic regression model was used to assess the predictors of LVH in postmenopausal women with hypertension. The best-fit nomogram was executed using R software. The calibration and decision curve were employed to verify the predictive accuracy of the nomogram. The results were evaluated in the validation set. *Results*: Menopause age (OR = 0.929, 95% CI 0.866–0.998, *p* = 0.044), BMI (OR = 1.067, 95% CI 1.019–1.116, *p* = 0.005), morning systolic blood pressure (SBP: OR = 1.050, 95% CI 1.032–1.069, *p* = 0.000), morning diastolic BP (DBP OR = 1.055, 95% CI 1.028–1.083, *p* = 0.003), angiotensin II receptor *blocker* (ARB) utilization rate (OR = 0.219, 95% CI 0.131–0.365, *p* = 0.000), LDL-C (OR = 1.460, 95% CI 1.090–1.954, *p* = 0.011) and cardio-ankle vascular index (CAVI) (OR = 1.415, 95% CI 1.139–1.757, *p* = 0.028) were associated with LVH in postmenopausal hypertension patients. The nomogram model was then developed using these variables. The internal validation trial showed that the nomogram model described herein had good performance in discriminating a C-index of 0.881 (95% CI: 0.837–0.924) and high quality of calibration plots. External validation of LVH-predictive nomogram results showed that the area under the ROC curve was 0.903 (95%CI 0.900–0.907). *Conclusions*: Our results indicate that the risk prediction nomogram model based on menopausal age, BMI, morning SBP, morning DBP, ARB utilization rate, LDL-C and CAVI has good accuracy and may provide useful references for the medical staff in the intuitive and individualized risk assessment in clinical practice.

## 1. Introduction

Hypertension has been identified as the main risk factor for cardiovascular disease (CVD) and also an important reason for the increased morbidity and mortality of CVD [1,2] It is estimated that various types of CVD account for about 30% deaths worldwide each year, and around 50% of these deaths are affected by CVD directly related to hypertension [3]. Left ventricular hypertrophy (LVH) is one of the most prominent manifestations of target organ damage in hypertensive patients, and an independent risk factor affecting the prognosis of hypertensive patients [4]. In the past few decades, immense researches have specifically focused on long-term pathological cardiac hypertrophy that can lead to arrhythmias, heart failure, and sudden cardiac death, increasing the risk of death [4,5].

Clinical studies have demonstrated that gender affects the formation and development of LVH [5,6]. The prevalence of hypertension and hypertension-mediated organ damage (HMOD) in premenopausal women is generally lower than that in men of the same age, but this gender advantage disappears rapidly after menopause [7]. In fact, menopause significantly increases the prevalence of hypertension and HMOD [8]. The incidence of LVH in postmenopausal women is much higher than that in men of the same age group [9].

Early screening of high-risk groups who may have LVH in postmenopausal hypertension patients and active formulation of intervention measures under the guidance of doctors are of great significance to improve the quality of life and long-term prognosis of postmenopausal women with hypertension. Several studies have focused on risk factors associated with the presence of LVH in postmenopausal women [10]. However, there is no exploratory study to assess the probability of future LVH risk in postmenopausal hypertension. Therefore, the purpose of this study is to establish a personalized nomogram model based on clinical data to predict the risk of LVH in Chinese postmenopausal hypertension, so as to guide the clinical screening of high-risk populations.

## 2. Methods

### 2.1. Trial Design and Participants

1486 postmenopausal hypertensive women aged between 49 and 68 years were recruited from Lanzhou University Second Hospital December 2017 to December 2019. Inclusion criteria were as follows: (1) the diagnosis of hypertension was determined by experienced physicians in accordance with 2010 Chinese guidelines for the management of hypertension diagnostic criteria for hypertension (systolic blood pressure ≥ 140 mmHg and/or diastolic blood pressure ≥ 90 mmHg) [11]; (2) menopause, which refers to the physiological phenomenon of complete ovarian failure and permanent cessation of menstruation in women with age (elevated blood pressure (BP) in women after one year of physical menopause is called postmenopausal hypertension) [12]; and (3) studies included participants with complete clinical data. Exclusion criteria included: (1) patients who were diagnosed with arterial hypertension before menopause; (2) heart valve disease, coronary artery disease, diabetes mellitus, hyperuricemia, secondary hypertension and heart valve disease, coronary artery disease and other cardiopathies; (2) ovarian hysterectomy; and (3) hepatic and renal dysfunction. After screening, 846 participants were allocated in a 2:1 ratio to either the training set (*n* = 550) or validation set (*n* = 284). Participants in the validation set were followed up for one year.

The sample size was estimated based on expected differences in the main outcome LVH. Based on our team’s preliminary research, sample size estimation was calculated for a sample power of 46%, with the allowable error of 4%, at the test level of 0.05 [13]. In this study, the permissible dropout rate was 20%.

This prospective study was reviewed and approved by the Ethics Review Committee of Lanzhou University Second Hospital (2018A-096).

### 2.2. Clinical Data Collection

General clinical data were collected including age, body mass index (BMI), educational level, smoking history, drinking history, family history of hypertension, history of antihypertensive drug use, morning systolic BP (SBP), and morning diastolic BP (DBP). Morning hypertension is measured after a patient wakes up without any activity, including taking antihypertensive medications. Morning hypertension is a home BP measurement. BMI was defined as weight divided by height squared (BMI < 18.50 kg/m^2^ (underweight = 1), 18.5–23.99 kg/m^2^ (normal = 2), 24–27.99 kg/m^2^ (overweight = 3), and ≥28.00 kg/m^2^ (obese = 4)) [14]. Morning BP refers to the home blood pressure monitoring results within 1 h after waking up in the morning (before taking medicine and breakfast) or the BP between 2 h after waking up or 6:00–10:00 in the morning recorded by the ambulatory BP monitoring [15].

### 2.3. Echocardiography

The Philips IE33 ultrasound system was used to measure the cardiac structure. The measurements were based on guidelines from the American Society of Echocardiography and the European Society of Cardiovascular Imaging. The mean values of three cardiac cycles were used as the final measurements for all the indexes. Reflecting cardiac structural indicators include left ventricular end-diastolic diameter (LVEDD), interventricular septal thickness (IVST), LV posterior wall thickness (LVPWT). LV mass index values (LVMi) were calculated by LVMI = 0.8 × 1.04 × [(LVEDD + IVST + LVPWT)3 − LVEDD3] + 0.6/body surface area. The diagnostic criteria for LVH were according to the 2018 ESC/ESH Guidelines for the management of arterial hypertension: LVMI ≥ 115 g/m^2^ (male) or ≥95 g/m^2^ (female). Based on the diagnostic criteria of female LVH, postmenopausal female hypertension patients were divided into an LVH group and a non-LVH group. The ultrasonic data of each group were measured repeatedly for 3 times by professionals. The average of the three measurements is taken as the final measurement result.

### 2.4. Laboratory Testing

Major laboratory indicators were tested in our central laboratories. The relevant indexes were determined by Cobas-8000 automatic biochemical analyzer (Roche Diagnostics, Mannheim, Germany).

### 2.5. Vascular Function

The MB3000 arteriosclerosis tester was used to examine vascular sclerosis, including cardio-ankle vascular index (CAVI) reflecting arterial elasticity and arteriosclerosis. After participants rested on their back for 15 min, trained technicians placed appropriately sized cuffs on the patient’s upper arms and ankles, respectively. Electrocardiogram electrodes were installed on both wrists and the heart sound sensor was attached to the sternum in the second intercostal space. The knee pulse sensor was mounted on the patient’s knee and the airbag was aligned with the center of the popliteal fossa. After entering the patient information, the machine automatically measured and calculated the CAVI value. CAVI = a [(2ρ/ΔP) × ln(Ps/Pd)PWV2] + b (ρ: blood viscosity, Ps: SBP, Pd: DBP, ΔP: Ps-Pd, PWV: pulse wave velocity).

### 2.6. Questionnaire Survey

In the form of face-to-face interview and questionnaire, the researchers filled out the survey records. The Mini-mental State Examination (MMSE) scale was used to preliminarily assess the participants’ intellectual status and degree of cognitive impairment [16]. Sexual function in postmenopausal women with hypertension was assessed by the Female Sexual Function Index (FSFI) scale [17]. The study was conducted by trained investigators according to strict form-filling explanations.

### 2.7. Follow-Up

Patients in the validation set were followed up for one year. At the end of the follow-up visit, the following contents were recorded: (1) general clinical characteristics; (2) laboratory, echocardiography, and vascular sclerosis examination results; and (3) result of interview and questionnaire. Patients lost to follow-up and with incomplete follow-up data were excluded from the study.

## 3. Statistical Analysis

R software and SPSS26.0 statistical software were used to process all the data. Continuous variables were tested for normal distribution by Kolmogorow–Smirnov test. Measurement data that conformed to the normal distribution were expressed as means ± standard deviation (SD), while those that did not conform to the normal distribution were presented as median. The categorical variables were expressed as values and percentages. Continuous variables were compared using the Student T test or the Mann–Whitney U test. The chi-square test was used to compare the classifying variables. Nonparametric tests were applicable to data that were not normally distributed.

Variables were subjected to multivariable logistic regression analysis to determine independent factors associated with LVH in postmenopausal hypertensive patients. Finally, a nomogram was established based on the identified independent risk factors. The method of Bootstrap repeated sampling was used for internal validation of the model, and the calibration curve was drawn to evaluate the consistency of the model. The C-index and AUC were used to evaluate the differentiation or accuracy of the model. The closer the C-index and AUC value to one, the more accurate the prediction ability of the column chart would be. We also used the decision curve to evaluate the nomogram. In addition, receiver operating characteristic curve (ROC) was drawn, and area under curve was calculated for external validation. *p* < 0.05 was considered statistically significant.

## 4. Results

### 4.1. Baseline Characteristics and Risk Factors of LVH Women with Postmenopausal Hypertension

**Figure 1:**
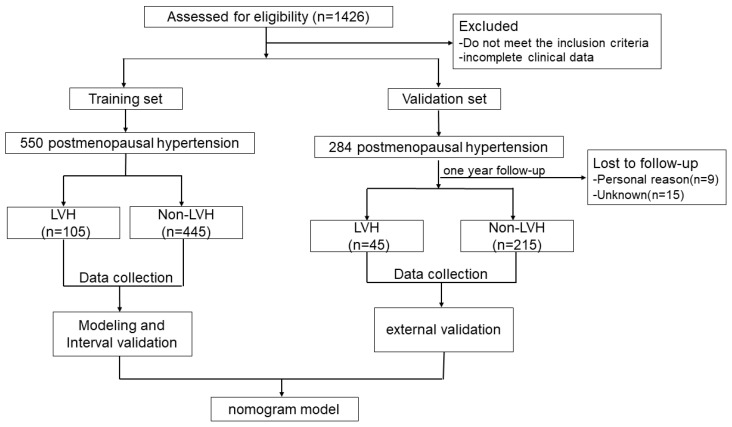
Research flow diagram; LVH: left ventricular hypertrophy.

From December 2017 to December 2019, 846 participants were screened for eligibility (Figure 1). In the training set, there were 105 LVH patients in the study population, with an incidence of 18.5%. In the validation set, 15 patients were lost to follow-up and 9 patients withdrew from the study for personal reasons during the one-year follow-up. 45 patients were eventually assigned to the LVH group and 215 to the non-LVH group. The result of univariate analysis indicated that, compared with the non-LVH group, the LVH group had higher morning BP, BMI, earlier menopause, higher angiotensin II type I receptor blockade (ARB) utilization rate, and lower MMSE score. There was no significant difference in waist-to-hip ratio, educational level and FSFI score between the two groups. All patients had normal left ventricular systolic function and ejection fraction. The analysis showed significant differences in low-density lipoprotein cholesterol (LDL-C), CAVI, LVEDD, IVST, LVPWT, and LVMI between the two groups. There was no statistical difference in the remaining indexes. The clinical data of the postmenopausal hypertensive patients with and without LVH are summarized in Table 1**.** The indicators of echocardiography and vascular function in the two groups are summarized in Table 2.

### 4.2. Establishment and Internal Validation of LVH-Predictive Nomogram

The results of multivariable logistic regression analysis showed that menopause age (OR = 0.929, 95% CI 0.866–0.998, *p* = 0.044), BMI (OR = 1.067, 95% CI 1.019–1.116, *p* = 0.005), morning SBP (OR = 1.050, 95% CI 1.032–1.069, *p* = 0.000), morning DBP (OR = 1.055, 95% CI 1.028–1.083, *p* = 0.003), ARB utilization rate (OR = 0.219, 95% CI 0.131–0.365, *p* = 0.000), LDL-C (OR = 1.460, 95% CI 1.090–1.954, *p* = 0.011) and CAVI (OR = 1.415, 95% CI 1.139–1.757, *p* = 0.028) were independent predictors of LVH in postmenopausal women with hypertension (Table 3).

**Figure 2:**
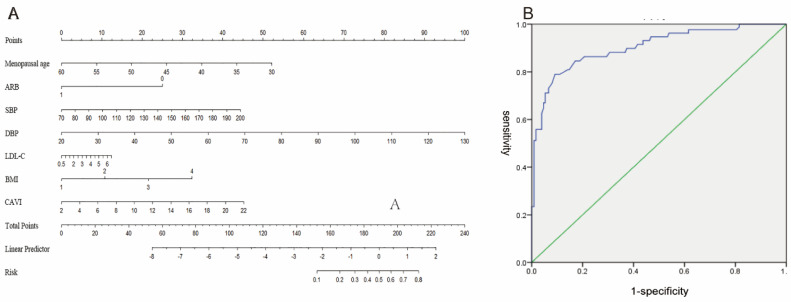
Modeling and external validation. (**A**): the nomogram of the risk model of left ventricular hypertrophy in patients with postmenopausal hypertension; (**B**): the ROC curve (external validation of LVH-predictive nomogram). ARB: angiotensin receptor blocker; BMI: body mass index; CAVI: cardio-ankle vascular index; DBP: diastolic blood pressure; LDL-C: low-density lipoprotein cholesterol; ROC curve: receiver operating characteristic curve; SBP: systolic blood pressure.

**Figure 3:**
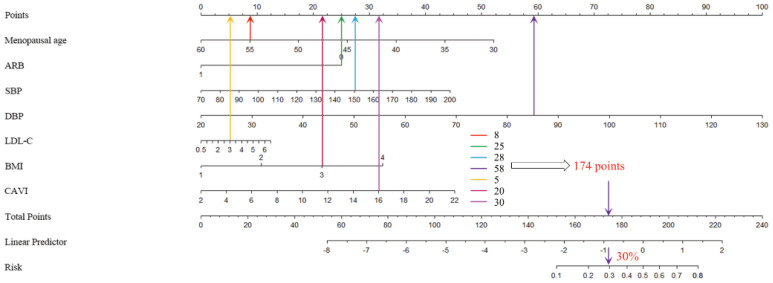
Case: a 55-year-old menopausal patient with hypertension (8 points) who did not take ARB drugs (25 points) had an early morning systolic blood pressure of 150 mmHg (28 points), diastolic blood pressure of 85 mmHg (58 points), LDL-C of 3 mmol/L (5 points), BMI greater than 24 kg/m^2^ (20 points), CAVI of 16 (30 points), corresponding total score of 174 points, and the predicted risk value of the nomogram was about 30%. ARB: angiotensin receptor blocker; BMI: body mass index; CAVI: cardio-ankle vascular index; DBP: diastolic blood pressure; SBP: systolic blood pressure; LDL-C: low-density lipoprotein cholesterol.

Based on the above analysis of independent predictors and professional knowledge, a nomogram was constructed to visualize the logistic regression model (Figure 2A). For example, a 55-year-old menopausal patient with hypertension (8 points) who did not take ARB drugs (25 points) had an early morning SBP of 150 mmHg (28 points), DBP of 85 mmHg (58 points), LDL-C of 3 mmol/L (5 points), BMI greater than 24 kg/m^2^ (20 points), CAVI of 16 (30 points), had a total score of 174 points; the predicted risk value using the nomogram was about 30% (Figure 3).

**Figure 4:**
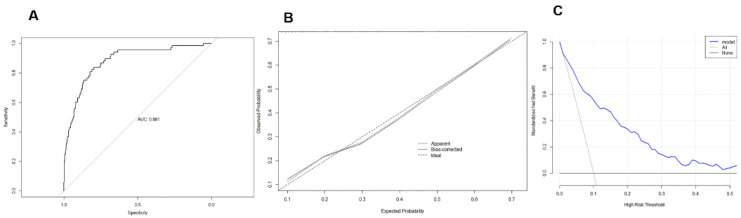
Internal validation of the model. (**A**): the receiver operating characteristic curve of prediction model of left ventricular hypertrophy in patients with postmenopausal hypertension; (**B**): calibration curve of prediction model of left ventricular hypertrophy in patients with postmenopausal hypertension; (**C**): decision curve of prediction model of left ventricular hypertrophy in patients with postmenopausal hypertension.

The C-index was used to evaluate the discrimination degree of the nomogram, namely concordance index (C-index) = 0.881 (95% CI 0.837–0.924), showing its good accuracy (area under the receiver operating characteristic curve (AUC) = C-index = 0.881) (Figure 4A). After the internal verification of the nomogram model by the method of Bootstrap repeated 1000 times sampling, a high-quality calibration curve of the prediction model was obtained, indicating that there was a good consistency between the prediction model and the actual observed results (Figure 4B).

In the decision curve, the X-axis represented threshold probability and the Y-axis represented net benefit. The solid blue line represents the nomogram’s prediction of LVH risk in postmenopausal women with hypertension, the solid gray line represents the hypothesis that all patients had exacerbation (all), and the solid black line represents the hypothesis that none of the patients had exacerbation (none), as shown in Figure 4C. The decision curve analysis showed that the use of this nomogram to assess the risk of LVH in postmenopausal women with hypertension had some significance.

### 4.3. External Validation of LVH-Predictive Nomogram

About 17.3% of postmenopausal hypertension patients developed LVH during a one-year follow-up in the validation set. The predictive value of the model for the future LVH of postmenopausal hypertension was analyzed by drawing the ROC curve. The results showed that the area under the ROC curve was 0.903 (95% CI 0.900–0.907) (Figure 2B).

## 5. Discussion

In this study, the nomogram based on clinical analysis was constructed to evaluate the risk of LVH in Chinese postmenopausal hypertensive women. The results of this study presented that menopause age, BMI, morning SBP, morning DBP, ARB utilization rate, LDL-C and CAVI were included in the prediction model. The nomogram showed good consistency and discrimination in predicting the risk of LVH in Chinese postmenopausal hypertensive patients. This tool can help clinicians identify high-risk patients with LVH in postmenopausal hypertensive women and initiate appropriate interventions without requiring complex medical examination.

Why not use the electrocardiogram (ECG) or echocardiography to assess LVH in postmenopausal women with hypertension? It is well known that the ECG has a low sensitivity for diagnosis of LVH. The sensitivity, specificity, positive predictive value and negative predictive value of ECG criteria for LVH were 12–29%, 93–96%, 62–71% and 67–71%, respectively [18]. In contrast, three-dimensional echocardiography was more accurate in evaluating LVH results compared with ECG, but has limited usability and high technical requirements. More importantly, ECG and echocardiography can only assess the LVH that has occurred, and cannot predict the possible risk of LVH in the future. However, the nomogram can visualize the results of complex logistics regression equations, and accurately predict the probability of an individual’s actual outcome event based on the value of the prediction parameters [19]. This tool can make the results of predictive models more readable, help clinicians to evaluate patients and formulate effective treatment strategies. At present, more and more studies have used the nomogram model to predict risk and prognosis of disease. In this study, a nomogram based on seven easily accessible items was used to assess the incidence of LVH in postmenopausal hypertensive women in China. The results of the calibration curve and the decision curve showed that the nomogram model had favorable recognition and calibration capabilities, which means that our nomogram may be widely used in clinical practice.

After menopause, the ovarian function fails and the estrogen level in the body undergoes significant change [20]. The level of circulating estrogen and estrone would decrease by more than 90% and 70%, respectively [21]. Clinical and basic studies have shown that estrogen deficiency will further promote the development and progression of LVH [22,23]. Therefore, the earlier the age of menopause, the less estrogen protects the cardiovascular system and the higher the risk of LVH. The incidence of obesity in postmenopausal women is as high as 40%, and it is often accompanied by the occurrence of metabolic syndrome [24]. The increase in body mass is proportional to the incidence of CVD [25]. In their study involving 30,920 individuals including 11,792 obese patients, Lavie et al. found that the incidence of LV geometric abnormality increased by 49% in obese subjects, 34% in patients with centripetal hypertrophy, and 7% in patients with eccentric hypertrophy, which is consistent with the finding of the present study that the lower the BMI, the lower the risk of LVH in postmenopausal women with hypertension [26]. Morning blood pressure was reported to be significantly related to LVH and LV diastolic function [27]. Target organ damage could still be observed in patients with morning hypertension who received antihypertensive therapy [28]. Shibamiya et al. found that the risk of LVH increased by 23% for every 10 mmHg increase in morning systolic blood pressure [29]. The results of this study also demonstrated that inadequate morning blood pressure control was the main reason for the increased risk of LVH in postmenopausal hypertension patients. In addition, this study also suggested that the use of antihypertensive drug ARB was associated with the risk of LVH in postmenopausal women with hypertension. The renin-angiotensin-aldosterone system is an important target for anti-hypertension and reversing LVH [30]. Compared with drugs that simply reduce the level of angiotensin II (AngII), such as angiotensin-converting enzyme inhibitors (ACEI), ARB can activate the ACE2-Ang (1–2)-Mas system by increasing the level of endogenous AngII to play a cardioprotective effect [31].

It is worth noting that the univariate analysis of this study revealed that compared with the non-LVH group, the LVH group had lower MMSE scores and more severe cognitive impairment. There is tropism evidence that LVH is independently associated with cognitive dysfunction. Scuteri et al. reported that the existence of LVH was negatively correlated with cognitive performance, implying that the higher the LVMI, the worse the MMSE score [32]. The possible mechanism linking LVH and cognitive dysfunction is the presence of cerebral white matter damage (WMD) [33]. Studies found that patients with cardiovascular risk factors had varying degrees of WMD in the dorsolateral prefrontal areas and the lateral orbitofrontal circuits [33,34]. However, the reason why MMSE cannot be a risk factor for predicting LVH in future may be that (1) the results of the study were affected by the cross-sectional design; (2) the limitations of cognitive ability assessment using a single MMSE reduced the sensitivity of detecting specific cognitive difficulties from vascular origin.

Based on the seven independent risk factors (menopausal age, BMI, morning blood pressure, ARB use of antihypertensive drugs, LDL-C and CAVI), this study established for the first time an individual nomogram model to predict the risk of LVH in Chinese postmenopausal women with hypertension. After menopause, the ovarian function fails and the estrogen level in the body undergoes significant change [20]. After menopause, the ovarian function fails and the estrogen level in the body undergoes significant change [20]. The level of circulating estrogen and estrone may decrease by more than 90% and 70%, respectively [21]. Therefore, the prevalence of the post-menopausal women’s hypertension and HMOD is significantly increased. Early screening of high-risk groups who may have HP in post-menopausal women and active formulation of intervention measures under the guidance of doctors are of great significance to improve the quality of life and long-term prognosis of postmenopausal women. Therefore, the purpose of this study is to establish a personalized nomogram model based on clinical data to predict the risk of LVH around the world in post-menopausal women, so as to guide the clinical screening of high-risk populations. It is of great significance for clinicians to identify high-risk groups and take more targeted prevention and treatment measures. However, the sample size in this study was small and the included influencing factors were limited and these data have not been verified in different populations outside China. These deficiencies limit the accuracy of the results, which can be further verified by multi-center and large sample data.

## 6. Conclusions

The present study’s results have a great impact on the prevention strategy of LVH in postmenopausal hypertension patients. Our results indicate that the risk prediction nomogram model based on menopausal age, BMI, morning SBP, morning DBP, ARB utilization rate, LDL-C and CAVI has good accuracy and may provide useful references for the medical staff in the intuitive and individualized risk assessment in clinical practice.

## Figures and Tables

**Table 1:** Baseline clinical characteristics of postmenopausal women with hypertension in the training and validation sets.

	Training Set (*n* = 550)			Validation Set (*n* = 260)		
	LVH (*n* = 105)	non-LVH (*n* = 445)	*p* Value	LVH (*n* = 45)	Non-LVH (*n* = 215)	*p* Value
menopausal age	47.69 ± 4.03	48.62 ± 3.26	0.012 *	57.1 ± 4.28	59.00 ± 3.38	0.001 *
BMI ≥ 24 (kg/m^2^)	64 (61)	129 (29)	<0.001	20 (44)	60 (28)	0.029 *
WHR	0.83 ± 0.07	0.84 ± 0.06	0.141	1 ± 0.001	1 ± 0.007	0.648
SBP (mmHg)	151.85 ± 15.03	141.58 ± 20.91	<0.001 *	154.56 ± 13.42	143.31 ± 18.74	<0.001
DBP (mmHg)	91.73 ± 13.24	82.86 ± 13.48	<0.001 *	94.96 ± 10.58	85.59 ± 13.58	<0.001
HR (beats/min)	81.95 ± 12.04	78.90 ± 11.48	0.016 *	82.40 ± 12.59	80.84 ± 11.89	0.430
Educational level (college degree or above), *n* (%)	38 (36)	176 (40)	0.348	18 (40)	95 (44)	0.606
Family history of hypertension, *n* (%)	67 (64)	258 (58)	0.250	30 (67)	135 (63)	0.623
ACEI, *n* (%)	36 (34)	114 (26)	0.122	6 (13)	67 (31)	0.016 *
ARB, *n* (%)	45 (43)	328 (74)	<0.001 *	19 (42)	140 (65)	0.004 *
BB, *n* (%)	24 (23)	91 (20)	0.585	10 (22)	55 (26)	0.636
CCB, *n* (%)	75 (71)	305 (69)	0.564	33 (73)	152 (71)	0.723
DIU, *n* (%)	25 (24)	128 (29)	0.308	9 (20)	57 (27)	0.316
FSFI score	17.45 ± 6.95	18.23 ± 7.78	0.347	16.84 ± 8.13	17.78 ± 7.11	0.434
MMSE score	26.26 ± 2.10	26.92 ± 2.22	0.006 *	25.84 ± 2.40	26.88 ± 1.95	0.002 *
Laboratory						
BUN (mmol/L)	4.85 ± 1.58	4.66 ± 1.30	0.195	4.78 ± 1.24	4.67 ± 1.24	0.596
CR (μmol/L)	60.27 ± 9.17	61.15 ± 9.78	0.400	59.44 ± 7.82	60.75 ± 9.58	0.391
TC (mmol/L)	4.69 ± 0.73	4.71 ± 0.88	0.825	4.62 ± 0.78	4.70 ± 0.83	0.590
TG (mmol/L)	1.74 ± 1.32	1.69 ± 0.95	0.698	3.00 ± 1.78	1.68 ± 1.03	0.530
LDL-C (mmol/L)	2.75 ± 0.75	2.56 ± 0.69	0.010 *	2.81 ± 0.82	2.54 ± 0.68	0.019 *
proteinuria, *n* (%)	5 (5)	13 (3)	0.340	5 (11)	13 (6)	0.224
Echocardiography						
LVEDD (mm)	43.74 ± 3.88	40.44 ± 2.93	<0.001	43.07 ± 2.98	41.15 ± 1.96	<0.001
IVST (mm)	11.58 ± 1.03	9.83 ± 0.78	<0.001	11.93 ± 1.00	10.44 ± 0.73	<0.001
LVPWT (mm)	11.63 ± 1.05	9.85 ± 0.77	<0.001	11.89 ± 0.82	10.50 ± 0.87	<0.001
LVMI (g/m^2^)	104.94 ± 9.71	73.13 ± 7.08	<0.001	108.78 ± 10.39	81.81 ± 5.57	<0.001
Vascular function						
CAVI (Right)	8.28 ± 0.98	7.99 ± 1.35	0.040 *	8.60 ± 0.81	8.14 ± 1.38	0.031 *
CAVI (Left)	8.23 ± 0.99	7.89 ± 1.22	0.008 *	8.44 ± 0.78	8.01 ± 1.12	0.015 *

Continuous variables were compared using the Student-T test or the Mann-Whitney U test. The chi-square test was used to compare the classifying variables. * *p* < 0.05 indicates a statistically significant differences among LVH and non-LVH. ACEI: angiotensin-converting enzyme inhibitor; ARB: angiotensin receptor blocker; BB: beta-blockers; BMI: body mass index; BUN: blood urea nitrogen; CCB: calcium channel blocker; CR: creatinine; CAVI: cardio-ankle vascular index; DBP: diastolic blood pressure; DIU: diuretic; FSFI: female Sexual Function Index; HR: heart rate; LVH: left ventricular hypertrophy; LDL-C: low-density lipoprotein cholesterol; MMSE: Mini-mental State Examination; RBC: red blood cell; SBP: systolic blood pressure; TC: total cholesterol; TG: triglyceride.

**Table 2:** Echocardiographic and vascular function indexes of the study population.

	LVH (*n* = 69)	Non-LVH (*n* = 678)	*p* Value
LVEF (%)	63.10 ± 5.49	64.10 ± 2.58	0.008 *
LVDd (mm)	43.70 ± 4.25	39.82 ± 2.74	0.000 *
IVSTd (mm)	11.65 ± 1.15	9.54 ± 1.01	0.000 *
LVPWTd (mm)	11.64 ± 1.18	9.55 ± 0.99	0.000 *
RVWT (mm)	0.54 ± 0.09	0.48 ± 0.06	0.000 *
LAD (mm)	35.09 ± 5.55	29.75 ± 2.80	0.000 *
LAVi (mL/m^2^)	21.46 ± 6.93	19.96 ± 3.28	0.002 *
LVMI (kg/m^2^)	106.19 ± 10.05	68.63 ± 11.91	0.000 *
E/e′	9.22 ± 3.42	7.91 ± 1.64	0.000 *
PASP (mmHg)	20.80 ± 8.96	19.94 ± 5.13	0.224

* *p* < 0.05 indicates a statistically significant differences among LVH and non-LVH. ABI: ankle-brachial index; CAVI: cardio-ankle vascular index; E/e′: the peak velocity at early diastole/mitral annulus mean velocities during early diastole; IVST: interventricular septal thickness; LAD: diameter of left atrium; LAVi: left atrial volume index; LVMi: left ventricular mass index; LVDd: left ventricular end-diastolic diameter; LVEF: left ventricular ejection fraction; LVPWT: left ventricular posterior wall thickness; PASP: pulmonary artery systolic pressure; RVWT: relative ventricular wall thickness.

**Table 3:** Logistic regression analysis of the risk of left ventricular hypertrophy in patients with postmenopausal hypertension.

Risk Factors	Partial Regression Coefficient	SE	OR	95% CI	*p* Value
menopausal age	−0.073	0.036	0.929	0.866–0.998	0.044 *
BMI	0.065	0.023	1.067	1.019–1.116	0.005 *
ARB	−1.521	0.262	0.219	0.131–0.365	0.000 *
morning SBP	0.049	0.009	1.050	1.032–1.069	0.000 *
morning DBP	0.054	0.013	1.055	1.028–1.083	0.003 *
LDL-C	0.378	0.149	1.460	1.090–1.945	0.011 *
CAVI	0.347	0.111	1.415	1.139–1.757	0.028 *

* *p* < 0.05 indicates a statistically significant differences. ARB: angiotensin receptor blocker; BMI: body mass index; CAVI: cardio-ankle vascular index; DBP: diastolic blood pressure; SBP: systolic blood pressure; SE: standard error.

## Data Availability

There is no new data were created.
